# Rare copy number variation in posttraumatic stress disorder

**DOI:** 10.1038/s41380-022-01776-4

**Published:** 2022-09-21

**Authors:** Adam X. Maihofer, Worrawat Engchuan, Guillaume Huguet, Marieke Klein, Jeffrey R. MacDonald, Omar Shanta, Bhooma Thiruvahindrapuram, Martineau Jean-louis, Zohra Saci, Sebastien Jacquemont, Stephen W. Scherer, Elizabeth Ketema, Allison E. Aiello, Ananda B. Amstadter, Esmina Avdibegović, Dragan Babic, Dewleen G. Baker, Jonathan I. Bisson, Marco P. Boks, Elizabeth A. Bolger, Richard A. Bryant, Angela C. Bustamante, Jose Miguel Caldas-de-Almeida, Graça Cardoso, Jurgen Deckert, Douglas L. Delahanty, Katharina Domschke, Boadie W. Dunlop, Alma Dzubur-Kulenovic, Alexandra Evans, Norah C. Feeny, Carol E. Franz, Aarti Gautam, Elbert Geuze, Aferdita Goci, Rasha Hammamieh, Miro Jakovljevic, Marti Jett, Ian Jones, Milissa L. Kaufman, Ronald C. Kessler, Anthony P. King, William S. Kremen, Bruce R. Lawford, Lauren A. M. Lebois, Catrin Lewis, Israel Liberzon, Sarah D. Linnstaedt, Bozo Lugonja, Jurjen J. Luykx, Michael J. Lyons, Matig R. Mavissakalian, Katie A. McLaughlin, Samuel A. McLean, Divya Mehta, Rebecca Mellor, Charles Phillip Morris, Seid Muhie, Holly K. Orcutt, Matthew Peverill, Andrew Ratanatharathorn, Victoria B. Risbrough, Albert Rizzo, Andrea L. Roberts, Alex O. Rothbaum, Barbara O. Rothbaum, Peter Roy-Byrne, Kenneth J. Ruggiero, Bart P. F. Rutten, Dick Schijven, Julia S. Seng, Christina M. Sheerin, Michael A. Sorenson, Martin H. Teicher, Monica Uddin, Robert J. Ursano, Christiaan H. Vinkers, Joanne Voisey, Heike Weber, Sherry Winternitz, Miguel Xavier, Ruoting Yang, Ross McD Young, Lori A. Zoellner, Psychiatric Genomics Consortium PTSD Working Group, Psychiatric Genomics Consortium CNV Working Group, Rany M. Salem, Richard A. Shaffer, Tianying Wu, Kerry J. Ressler, Murray B. Stein, Karestan C. Koenen, Jonathan Sebat, Caroline M. Nievergelt

**Affiliations:** 1 Department of Psychiatry, University of California San Diego, La Jolla, CA USA; 2 Department of Family Medicine and Public Health, University of California San Diego, La Jolla, CA USA; 3 Veterans Affairs San Diego Healthcare System, Center of Excellence for Stress and Mental Health, San Diego, CA USA; 4 The Hospital for Sick Children, Genetics and Genome Biology, Toronto, Ontario Canada; 5 The Hospital for Sick Children, The Centre for Applied Genomics, Toronto, Ontario Canada; 6 Centre Hospitalier Universitaire Sainte-Justine Centre de Recherche, Montreal, Quebec Canada; 7 Bioinformatics and Systems Biology Graduate Program, University of California San Diego, La Jolla, CA USA; 8 Department of Pediatrics, Centre Hospitalier Universitaire Sainte-Justine Centre de Recherche, Montreal, Quebec Canada; 9 Department of Genetics, Centre Hospitalier Universitaire Vaudois, Lausanne, Vaud Switzerland; 10 Department of Pediatrics, University of Montreal, Montreal, Quebec Canada; 11 University of Toronto, McLaughlin Centre, Toronto, Ontario Canada; 12 Department of Molecular Genetics, University of Toronto, Toronto, Ontario Canada; 13 Research Service, Veterans Affairs San Diego Healthcare System, San Diego, CA USA; 14 Department of Epidemiology, Robert N Butler Columbia Aging Center, Columbia University, New York, NY USA; 15 Department of Psychiatry, Virginia Institute for Psychiatric and Behavioral Genetics, Richmond, VA USA; 16 Department of Psychiatry, University Clinical Center of Tuzla, Tuzla, Bosnia and Herzegovina; 17 Department of Psychiatry, University Clinical Center of Mostar, Mostar, Bosnia and Herzegovina; 18 Psychiatry Service, Veterans Affairs San Diego Healthcare System, San Diego, CA USA; 19 MRC Centre for Psychiatric Genetics and Genomics, Cardiff University, National Centre for Mental Health, Cardiff, South Glamorgan UK; 20 Department of Psychiatry, UMC Utrecht Brain Center, Utrecht, the Netherlands; 21 Department of Psychiatry, Harvard Medical School, Boston, MA USA; 22 McLean Hospital, Belmont, MA USA; 23 Department of Psychology, University of New South Wales, Sydney, NSW Australia; 24 Division of Pulmonary and Critical Care Medicine, Department of Internal Medicine, University of Michigan Medical School, Ann Arbor, MI USA; 25 CEDOC-Chronic Diseases Research Centre, Lisbon Institute of Global Mental Health, Lisbon, Portugal; 26 Lisbon Institute of Global Mental Health and Comprehensive Health Research Centre, Universidade Nova de Lisboa, Lisboa, Portugal; 27 University Hospital of Wuerzburg, Center of Mental Health, Psychiatry, Psychosomatics and Psychotherapy, Wuerzburg, Germany; 28 Department of Psychological Sciences, Kent State University, Kent, OH USA; 29 Research and Sponsored Programs, Kent State University, Kent, OH USA; 30 Department of Psychiatry and Psychotherapy, Medical Center-University of Freiburg, Faculty of Medicine, Freiburg, Germany; 31 Faculty of Medicine, Centre for Basics in Neuromodulation, University of Freiburg, Freiburg, Germany; 32 Department of Psychiatry and Behavioral Sciences, Emory University School of Medicine, Atlanta, GA USA; 33 Department of Psychiatry, University Clinical Center of Sarajevo, Sarajevo, Bosnia and Herzegovina; 34 Department of Psychological Sciences, Case Western Reserve University, Cleveland, OH USA; 35 Walter Reed Army Institute of Research, Medical Readiness Systems Biology, Center for Military Psychiatry and Neuroscience, Silver Spring, MD USA; 36 Netherlands Ministry of Defence, Brain Research and Innovation Centre, Utrecht, the Netherlands; 37 Department of Psychiatry, UMC Utrecht Brain Center Rudolf Magnus, Utrecht, the Netherlands; 38 Department of Psychiatry, University Clinical Centre of Kosovo, Prishtina, Kosovo; 39 Department of Psychiatry, University Hospital Center of Zagreb, Zagreb, Croatia; 40 US Medical Research & Development Comm, Fort Detrick, MD USA; 41 Walter Reed Army Institute of Research, Headquarter, Silver Spring, MD USA; 42 Department of Health Care Policy, Harvard Medical School, Boston, MA USA; 43 Ohio State University, College of Medicine, Institute for Behavioral Medicine Research, Columbus, OH USA; 44 School of Biomedical Sciences, Queensland University of Technology, Kelvin Grove, QLD Australia; 45 Department of Psychiatry and Behavioral Sciences, Texas A&M University College of Medicine, Bryan, TX USA; 46 Institute for Trauma Recovery, University of North Carolina at Chapel Hill, Chapel Hill, NC USA; 47 Department of Translational Neuroscience, UMC Utrecht Brain Center Rudolf Magnus, Utrecht, the Netherlands; 48 Department of Psychological & Brain Sciences, Boston University, Boston, MA USA; 49 Department of Psychiatry, University Hospitals, Cleveland, OH USA; 50 Department of Psychology, Harvard University, Boston, MA USA; 51 Department of Emergency Medicine, UNC Institute for Trauma Recovery, Chapel Hill, NC USA; 52 Queensland University of Technology, Centre for Genomics and Personalised Health, Kelvin Grove, QLD Australia; 53 Gallipoli Medical Research Foundation, Greenslopes Private Hospital, Greenslopes, QLD Australia; 54 Walter Reed Army Institute of Research, Silver Spring, MD USA; 55 Department of Psychology, Northern Illinois University, DeKalb, IL USA; 56 Department of Psychology, University of Washington, Seattle, WA USA; 57 Department of Epidemiology, Columbia University Mailmain School of Public Health, New York, NY USA; 58 Department of Epidemiology, Harvard T. H. Chan School of Public Health, Boston, MA USA; 59 University of Southern California, Institute for Creative Technologies, Los Angeles, CA USA; 60 Department of Environmental Health, Harvard T.H. Chan School of Public Health, Boston, MA USA; 61 Department of Psychiatry and Behavioral Sciences, Medical University of South Carolina, Charleston, SC USA; 62 Department of Psychiatry and Behavioral Sciences, Emory University, Atlanta, GA USA; 63 Department of Psychiatry and Behavioral Sciences, University of Washington, Seattle, WA USA; 64 Department of Nursing and Department of Psychiatry, Medical University of South Carolina, Charleston, SC USA; 65 Department of Psychiatry and Neuropsychology, Maastricht Universitair Medisch Centrum, School for Mental Health and Neuroscience, Maastricht, Limburg the Netherlands; 66 University of Michigan, School of Nursing, Ann Arbor, MI USA; 67 Department of Obstetrics and Gynecology, University of Michigan, Ann Arbor, MI USA; 68 Department of Women’s and Gender Studies, University of Michigan, Ann Arbor, MI USA; 69 University of Michigan, Institute for Research on Women and Gender, Ann Arbor, MI USA; 70 Developmental Biopsychiatry Research Program, McLean Hospital, Belmont, MA USA; 71 Genomics Program, College of Public Health, University of South Florida, Tampa, FL USA; 72 Department of Psychiatry, Uniformed Services University, Bethesda, MD USA; 73 Amsterdam Neuroscience, Mood, Anxiety, Psychosis, Sleep & Stress Program, Amsterdam, the Netherlands; 74 Department of Psychiatry, Amsterdam UMC Location Vrije Universiteit Amsterdam, Amsterdam, the Netherlands; 75 Department of Anatomy and Neurosciences, Amsterdam UMC Location Vrije Universiteit Amsterdam, Amsterdam, the Netherlands; 76 Universidade Nova de Lisboa, Nova Medical School, Lisboa, Portugal; 77 Queensland University of Technology, School of Clinical Sciences, Kelvin Grove, QLD Australia; 78 University of the Sunshine Coast, The Chancellory, Sippy Downs, QLD Australia; 79 University of California San Diego, School of Public Health, La Jolla, CA USA; 80 Broad Institute of MIT and Harvard, Stanley Center for Psychiatric Research, Cambridge, MA USA; 81 Department of Epidemiology, Harvard T. H. School of Public Health, Boston, MA USA; 82 Psychiatric and Neurodevelopmental Genetics Unit (PNGU), Massachusetts General Hospital, Boston, MA USA; 83 Cold Spring Harbor Laboratory, Cold Spring Harbor, NY USA; 84 Department of Cellular and Molecular Medicine, University of California San Diego, La Jolla, CA USA; 85 University of California San Diego, Herbert Wertheim School of Public Health and Human Longevity Science, La Jolla, CA USA; 86 Department of Epidemiology and Health Sciences, Naval Health Research Center, San Diego, CA USA; 87 Division of Epidemiology and Biostatistics, San Diego State University, School of Public Health, San Diego, CA USA; 88 University of California, San Diego, Moores Cancer Center, San Diego, CA USA

**Keywords:** Genetics, Psychiatric disorders, Predictive markers

## Abstract

Posttraumatic stress disorder (PTSD) is a heritable (*h*^2^ = 24–71%) psychiatric illness. Copy number variation (CNV) is a form of rare genetic variation that has been implicated in the etiology of psychiatric disorders, but no large-scale investigation of CNV in PTSD has been performed. We present an association study of CNV burden and PTSD symptoms in a sample of 114,383 participants (13,036 cases and 101,347 controls) of European ancestry. CNVs were called using two calling algorithms and intersected to a consensus set. Quality control was performed to remove strong outlier samples. CNVs were examined for association with PTSD within each cohort using linear or logistic regression analysis adjusted for population structure and CNV quality metrics, then inverse variance weighted meta-analyzed across cohorts. We examined the genome-wide total span of CNVs, enrichment of CNVs within specified gene-sets, and CNVs overlapping individual genes and implicated neurodevelopmental regions. The total distance covered by deletions crossing over known neurodevelopmental CNV regions was significant (beta = 0.029, SE = 0.005, *P* = 6.3 × 10^−8^). The genome-wide neurodevelopmental CNV burden identified explains 0.034% of the variation in PTSD symptoms. The 15q11.2 BP1-BP2 microdeletion region was significantly associated with PTSD (beta = 0.0206, SE = 0.0056, *P* = 0.0002). No individual significant genes interrupted by CNV were identified. 22 gene pathways related to the function of the nervous system and brain were significant in pathway analysis (FDR *q* < 0.05), but these associations were not significant once NDD regions were removed. A larger sample size, better detection methods, and annotated resources of CNV are needed to explore this relationship further.

## Introduction

Posttraumatic stress disorder (PTSD) has a substantial genetic component [1]. Recent large investigations of PTSD genetics have focused on common genetic variation [2, 3], but rare and structural forms of genetic variation are hypothesized to be important contributors to the development of psychiatric disorders [4]. Rare and structural variation have not received substantial empirical study in the context of PTSD [5]. However, these forms of variation have been examined more thoroughly in association with other psychiatric disorders, where many investigations have specifically focused on copy number variants (CNVs) [6]. CNV associations have been identified for attention-deficit/hyperactivity disorder (ADHD) [7], autism spectrum disorder (ASD) [8], depression [9, 10], obsessive-compulsive disorder [11], and schizophrenia [12]. Many of the identified psychiatric associations involved specific CNVs that have been implicated in neurodevelopmental disorders (NDD) [9, 10, 13], but also the cumulative burden of CNVs across the genome and enrichment over specific pathways related to the brain and development of the nervous system [12]. Largely owing to lack of available data, there has been no major reported investigation of CNVs and PTSD. However, the recent availability of large sample size PTSD genetic data [2] and available techniques to leverage this data to identify CNVs [14], means that it is now possible to investigate the association between PTSD and CNV burden with an unprecedented level of discovery power.

We present an association study between CNVs and PTSD, conducted in a sample of 114,383 (13,036 cases and 101,347 controls) European ancestry participants from the Psychiatric Genomics Consortium—PTSD [2, 15]. We detected rare (<1% population frequency) CNVs using algorithms [16–18] applied to the SNP genotyping array intensity data. Following this, we examined the impact of CNV on PTSD on multiple scales: genome-wide CNV burden, enrichment over 46 neuropsychiatric gene-sets [15], CNV burden on individual genes, and CNV carrier status over 53 previously implicated NDD CNV regions [9]. We conclude by comparing the risk contribution from CNVs to the contribution of common variant polygenic risk scores (PRSs).

## Methods

### Participants and phenotyping

The study sample consisted of 114,383 (13,036 cases and 101,347 controls) participants across 20 cohorts from the Psychiatric Genomics Consortium—PTSD freeze 2 data collection. The Psychiatric Genomics Consortium for PTSD is a large scale international effort to investigate genomic contributions to PTSD via meta-analysis of diverse cohorts [2]. For a given PGC-PTSD freeze 2 cohort to be included in this investigation, genotype intensity data had to be available, so that CNV calling could be performed. To reduce the potential for population stratification, we only included subjects of genetically determined [2] European ancestry, the largest homogeneous subset of the data. Within each cohort, participants were assessed for PTSD using either clinical assessment, clinician administered inventory, or self-reported inventory (Supplementary Table 1). Methods of PTSD assessment varied across cohort, and included the BSSS, CAPS, DEQ, IES, NSA, NWS, PCL, PSS, SCID, TSQ, and WMH-CIDI. All cohorts provided a PTSD case/control status variable as determined from standard criteria. Where applicable, PTSD symptom scores were computed for each inventory following inventory specific protocols for symptom scoring. All participants provided written informed consent, and studies were approved by the relevant institutional review boards and the University of California San Diego Human Research Protection Program.

### CNV detection

DNA was extracted from blood samples. All details regarding DNA extraction and genotyping processes have been published [2]. Participants were genotyped using Illumina arrays (Supplementary Table 1), with the exception that the UK Biobank (UKBB) cohort, which used Axiom genotyping arrays (ThermoFisher). Illumina genotype platform data was self-clustered in Genome-Studio 2.0 and exported as intensity data inputs for CNV callers (SNP name, chromosome, position, allele 1, allele 2, B allele frequency, log R ratio, X, and Y). Affymetrix platform genotype data clustering methods have been described previously [9], and log R ratio and B allele frequency data were downloaded directly from the UKBB database. For Illumina data, CNVs were called using PennCNV [17] and iPattern [16]. For Affymetrix data, CNVs were called using PennCNV and QuantiSNP [18]. For PennCNV calling, the population frequency of B allele files were generated using the data itself. Waviness correction was applied using a GC content model file generated from UCSC gc_model data (https://genome.ucsc.edu/cgi-bin/hgTables). For the Hidden Markov Model input of PennCNV, the pre-supplied files were used: hhall.hmm for Illumina data and affygw6.hmm for the UKBB data (https://penncnv.openbioinformatics.org/en/latest/user-guide/input/#hmm-file). iPattern calls were made using the default program settings, in batches of up to 196 samples. Batches were selected such that samples within a batch were genotyped on the same plate or genotyped at approximately the same time. QuantiSNP calls were made with 10 iterations of the EM algorithm, where the characteristic length used to calculate transition probabilities was set to 2,000,000. GC based correction was performed using UCSC gc_model files.

### CNV quality control

CNV were quality controlled according to the PGC CNV calling pipeline [12]. To ensure that the analysis included a reliable set of calls, CNV calls produced by the different calling algorithms were intersected to produce a consensus set. CNVs called as gain by one method and loss by the other were also excluded from further analyses. Fragmented large CNVs in a locus were annealed if the gap length between them was less than 30% of the overall length of the annealed CNV. CNV quality metrics calculated by PennCNV were used to perform sample QC. Subjects were removed if their values for SD of log R ratio, B allele frequency, or waviness were > = Q3 + 3IQR, if >20% of any chromosome was copy number variant (aneuploidy), or if they had excessive CNV count (≥Q3 + 3IQR CNVs) or KB burden (≥Q3 + 3 IQR megabases). Participants who failed standard genotype QC were removed: sample missingness rates > 2%, excess heterozygosity, mismatch between self-reported sex and genetically determined sex, π relatedness coefficient > 0.2. We removed CNVs for any of the following reasons: 50% overlap with centromeres, telomere, immunoglobulin or T-cell receptor loci, >50% overlap with known segmental duplications, CNV frequency >1% (measured within the data) in cases and controls and <10 kb in CNV length or intersecting <10 probes.

### CNV burden calculation

CNV burden was measured and evaluated for association with PTSD in multiple ways: The cumulative burden of CNVs was calculated as the genome-wide total distance (in megabases) spanned by CNVs. For each of the 53 NDD CNV regions, NDD CNV carrier status was determined as having at least 50% of the NDD CNV region overlapped by CNV. As a sensitivity analysis, two different overlap criteria (>0% or 100% overlap) were also evaluated. For gene-level CNV burden, first gene positions (GRCh37 human genome build) were downloaded from the UCSC table browser (https://genome.ucsc.edu/cgi-bin/hgTables). Genes were filtered to protein coding genes, based on having an “NM_” accession prefix in the National Center for Biotechnology Information reference sequence database [19]. For genes with multiple isoforms, the minimum start and the maximum end positions were used. For each CNV, the CNV was mapped to all genes it overlapped by at least one base pair. The CNV burden variable was then calculated for each gene, coded 1 if the subject carried a CNV that mapped onto the gene, and 0 otherwise. For gene-set analysis, a gene-set burden variable was calculated for each set tested, coded as the number of genes within the set overlapped by the CNVs. The gene-set analysis included 53 gene-sets, consisting of 23 gene-sets related to neurofunction or nervous system, 6 brain expression from BrainSpan consortium and 7 mouse phenotype negative control gene-sets from previous neurological disorders studies [12, 20], a set of loss-of-function intolerant genes as defined by gnomAD v2.0 [21], and 16 brain-expressed gene-sets from human neocortex scRNA data [22]. A list of genes belonging to each set is included in Supplementary Table 2.

### Statistical analyses

A two stage meta-analytic approach was conducted, where analyses were performed within each cohort separately then results were combined via meta-analysis. As all subjects belonging to a given cohort were genotyped using the same platform, this analysis was effectively performed stratified by platform, thus accounting for potential confounding due to CNV calling across platforms. Within each cohort, the association between PTSD and CNVs was tested using a regression model of PTSD as predicted by the CNV variable, five principal components calculated from genotype call data using Eigenstrat 6.0.2 [2] [23], and the log R ratio standard deviation sample quality metric from PennCNV. For the gene-set analyses, in order to follow the enrichment test model outlined by Raychaudhuri et al. [24] analyses also contained predictors for genome-wide total CNV count and genome-wide average length of CNVs. Cohorts with continuous PTSD symptom measures were analyzed using linear regression and cohorts with only case/control phenotypes were analyzed using logistic regression. Results across cohorts were meta-analyzed using fixed effects inverse variance weighted meta-analysis in the metafor [25] R package. For the meta-analysis, to account for the different PTSD measure scales used across cohorts, PTSD measures were scaled from 0 to 1 according to the theoretical range of scores of the assessment method (i.e., 0 = no PTSD symptoms, 1 = theoretical maximum possible PTSD symptoms), and case/control estimates were interpreted as being the observed, censored variable for a latent symptom measure variable. Statistical significance was declared based on Benjamini-Hochberg false discovery rate (FDR) *q* value < 0.05 calculated within a family of tests. To enhance interpretability of results, we also provide odds ratio effect estimates, via analyzing cohorts with continuous data using an ordinal logistic regression. For this analysis, odds ratio estimates were directly meta-analyzed across studies (i.e., not rescaled) using inverse-variance weighted meta-analysis. The statistical inferences made in this manuscript are however based only on the linear regression based results. To examine if outliers strongly contributed to the results of analyses of the 16p11.2 deletion and 2q13 deletion CNVs, linear regression was also performed using heteroskedasticity consistent (HC3) standard errors [26].

We estimated PRS for PTSD in all participants. SNP weights were obtained from the Million Veteran Program PTSD GWAS [3] of European ancestry participants, with weights adjusted using PRS-CS [27] under default parameters, with 1000 Genomes Phase 3 European data [28] used to model linkage disequilibrium. SNPs were filtered to common (minor allele frequency > 1%), strand unambiguous variants. PRS were computed as the weighted sum of risk alleles at each markers using the -score option in PLINK [29]. PRS were standardized to mean zero and unit standard deviation, such that the effects reported refer to PTSD risk relative to every unit standard deviation increase in PRS. The proportion of variance in PTSD explained by PRS and CNV was estimated as the difference in model r-squared values between a baseline model that included all relevant covariates and the model with additional terms for PRS and CNV. Standard errors for the proportion of variance explained were calculated using the formulae from Cohen et al. [30].

## Results

The PTSD CNV meta-analysis included 114,383 participants (13,036 cases and 101,347 controls) of European ancestry across 20 cohorts (Supplementary Table 1, Table 1). The method of PTSD assessment varied across cohorts (11 distinct methods), with most participants being assessed via a version of the PCL (*N* = 106,353). The majority of subjects (*N* = 113,320, 99%) were analyzed using PTSD symptom scores, the remaining subjects were analyzed using case/control status. 15 cohorts were genotyped using the Psych array (*N* = 6,813 samples), 1 with the Psych Chip (*N* = 756 samples), 3 with the OmniExpressExome+Custom content (*N* = 9432 samples), and 1 with the Affymetrix UK Biobank Axiom array (*N* = 97,382). CNVs were produced as the consensus call of iPattern and PennCNV (Illumina arrays, *N* = 19 studies) or PennCNV and QuantiSNP (Affymetrix array, *N* = 1). The final dataset included 103,036 CNVs (41,473 gains and 61,563 losses). The median length of CNVs was 122,756 BP (range=10,000 to 9,911,819 BP) (Supplementary Fig. 1). 60.1% of participants were carriers of at least one CNV (Table 1). Among CNV carriers, the average total span of CNV carried was 0.32 megabases (SD = 0.35), and the average of within subject average CNV lengths was 0.23 megabases (SD = 0.26).

**Table 1:** Cohorts analyzed.

Number	Study	Genotyping array	*N* subjects	*N* cases	*N* Controls	*N* CNV Carriers	*N* CNVs detected	*N* Deletions	*N* Duplications	CNV Span	SD	CNVs per Carrier	SD	Avg Length	SD
1	MRSC	OmniExpressExome8 + Custom	1374	149	1225	1147	2927	1399	1528	0.26	0.32	2.55	1.74	0.10	0.12
14	NSS1	OmniExpressExome8 + Custom	3664	890	2774	3031	7363	3308	4055	0.31	0.42	2.43	1.66	0.12	0.15
16	PPDS	OmniExpressExome8 + Custom	4394	781	3613	3649	9012	4052	4960	0.28	0.37	2.47	1.67	0.11	0.16
17	PTS1	PsychArray 1.1	364^b^	215	149	271	556	214	342	0.32	0.48	2.02	1.32	0.15	0.18
21	PSY2	PsychArray 1.1	4446	2226	2220	2997	6061	2929	3132	0.43	0.90	2.02	1.59	0.20	0.27
37	PSY3	PsychArray 1.1	699^a^	254	445	491	1017	537	480	0.37	0.53	2.07	1.57	0.18	0.23
41	NCMH	PsychArray 1.1	961	460	501	598	1067	442	625	0.43	0.67	1.78	1.08	0.23	0.31
52	FTCB	PsychChip	756	59	697	504	991	450	541	0.31	0.42	1.97	1.39	0.16	0.19
54	GRAC	PsychArray 1.1	151	66	85	99	174	91	83	0.29	0.31	1.76	1.11	0.16	0.14
55	GMRF	PsychArray 1.1	192	95	97	105	177	80	97	0.40	0.56	1.69	0.96	0.21	0.19
60	UKBB	Affymetrix UK Biobank Axiom array	97,382	7841	89,541	55,872	73,691	48,061	25,630	0.36	0.57	1.32	0.56	0.27	0.39

*Number* study accession number in PGC-PTSD Freeze 2, *N* sample size, *CNV Span* mean genomic length spanned by CNV (in megabases), in CNV carriers only, *N CNV* mean number of detected CNVs, among CNV carriers only; *Avg Length* across carriers, the mean of subjects’ mean CNV length (in megabases).

^a^Analyzed as a case/control study with 254 cases and 445 controls.

^b^Analyzed as a case/control study with 215 cases and 149 controls.

### Genome-wide CNV burden analysis

**Fig. 1:**
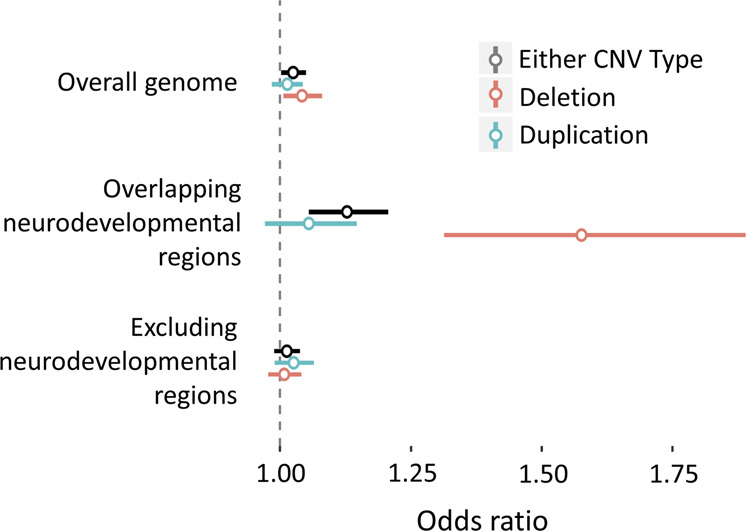
Genome-wide CNV burden association. The bar plot depicts regression beta coefficients as effect sizes (on the *x*-axis) of genome-wide CNV burden on PTSD, including overall burden, overlapping neurodevelopmental regions only, and genome-wide with neurodevelopmental regions excluded (on the *y*-axis). Data are shown stratified by CNV type, both CNV types (colored black), duplications only (colored red), and deletions only (colored blue). Effect sizes are shown in terms of megabases of the genome spanned by CNV.

Genome-wide cumulative CNV burden was significantly associated with PTSD (beta = 0.0028, SE = 0.0008, *P* = 0.0003, *q* = 0.001; OR = 1.025, 95%CI = [1.002,1.049]) (Fig. 1). We examined CNV burden stratified by type (duplication or deletion), finding that the total distance covered by deletions was significant (beta = 0.0046, SE = 0.0013, *p* = 0.0004, *q* = 0.001; OR = 1.042, 95%CI = [1.007,1.080]) but the total distance covered by duplications was not (beta = 0.0018, SE = 0.0010, *p* = 0.065, *q* = 0.11; OR = 1.054, 95%CI = [0.985–1.043]). Next, we examined CNV burden stratified by overlap with any of 53 previously implicated NDD CNV regions. The cumulative burden of CNV deletions that overlapped NDD regions was significantly associated with PTSD (beta = 0.0290, SE = 0.0054, *p* = 6.3 × 10^−8^, *q* = 1 × 10^−6^; OR = 1.576, 95% CI = [1.314,1.889]), while the duplication burden was not (beta = 0.0053, SE = 0.0023, *p* = 0.024, *q* = 0.06; OR = 1.055, 95%CI = [0.972,1.146]). The genome-wide burden of non-NDD CNV deletions was not significant (beta = 0.0031, SE = 0.0013, *p* = 0.023, *q* = 0.054; OR = 1.008,95%CI = 0.978–1.040) (Supplementary Table 3).

### Specific NDD CNV regions confer risk for PTSD

**Fig. 2:**
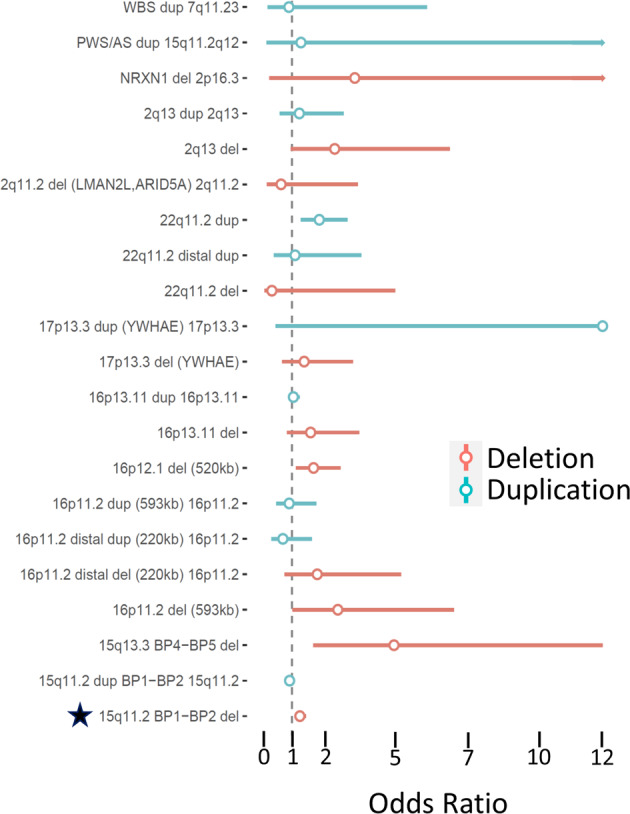
Association of individual NDD CNVs with PTSD. The bar plot depicts regression beta coefficients as effect sizes (on the *x*-axis) of NDD CNVs (on the *y*-axis) on PTSD. Data are colored by CNV type, with deletions in blue and duplications in red. Effect sizes are shown in terms of megabases of the genome spanned by CNV. A star indicates an FDR significant CNVs.

We investigated the association between PTSD and NDD CNV carrier status. 33 out of 53 NDD CNVs had at least 1 carrier (Supplementary Table 4). The most common NDD CNV was the 15q11.2 BP1-BP2 deletion (*N* = 529 carriers, frequency = 0.46%). Three NDD CNV were significantly associated with increased PTSD symptoms, the 2q13 deletion (chr2:111,394,040–112,012,649, *N* = 15 carriers, beta = 0.1455, SE = 0.0367, *p* = 0.0001, *q* = 0.0027; OR = 2.508, 95%CI = [0.956,6.583]), the 15q11.2 BP1‐BP2 microdeletion (chr15:22,805,313–23,094,530, *N* = 529 carriers, beta = 0.0206, SE = 0.0056, *p* = 0.0002, *q* = 0.0027; OR = 1.275, 95%CI = [1.093,1.488]), and the 16p11.2 deletion (*N* = 16 carriers, beta = 0.0702, SE = 0.025, *p* = 0.0041, *q* = 0.0369; OR = 2.619, 95%CI = [1.019,6.728]) (Fig. 2). Given the limited number of carriers for 2q13 deletion and 16p11.2 deletion, we tested their association again under models with robust standard errors, finding that the neither the 2q13 deletion nor the 16p11.2 deletion were significant (*p* = 0.11 and *p* = 0.25, respectively). The overall results were similar under a stricter definition of carrier status (100% overlap of NDD CNV region) (Supplementary Table 4), whereas under a loose definition of carrier status (>0% overlap of NDD CNV region), four regions were FDR significant: the 8p23.1 del (beta = 0.0233, SE = 0.0078, *p* = 0.003, *q* = 0.04; OR = 1.271, 95%CI = [1.021,1.582]), 15q11.2 BP1-BP2 del (beta=0.0201, SE = 0.0056, *p* = 0.0003, *q* = 0.007; OR = 1.27, 95%CI = [1.090,1.480]), 15q11.2-q12 Prader-Willi/Angelman syndrome del (beta = 0.0186, SE = 0.0053, p = 0.0004, q = 0.007; OR = 1.25, 95%CI = [1.080,1.447]), and 22q11.2 dup (beta = 0.0216, SE = 0.0055, *p* = 8.3 × 10^−5^, *q* = 0.0041; OR = 1.277, 95%CI = [1.128,1.444]). We note that in this less restrictive analysis, the association of the 15q11.2-q12 (Prader-Willi/Angelman syndrome) was driven by the smaller 15q11.2 BP1-BP2 deletion and that no subjects in this study carried a deletion with a >50% overlap with the Prader-Willi/Angelman syndrome critical region.

### Gene and gene-set analyses

We examined CNV association on the level of protein coding genes. 2880 genes harbored CNV with at least 0.01% frequency. We found that no gene was significant after multiple comparisons correction for the number of genes, in any strata (overall CNV, duplications, or deletions) (Supplementary Table 5). Following this we examined if CNV burden association with PTSD was enriched in any of 46 different gene-sets related to the brain and nervous system and 7 control gene-sets of non-brain tissue types. No control gene-set was significant. In contrast, 22 out of 46 sets related to the brain and nervous system were enriched in deletions (FDR *q* < 0.05) (Supplementary Table 6). Many of the top ranked genes in the significant sets overlapped with NDD CNV regions (Supplementary Table 7). As a sensitivity analysis, we removed CNVs overlapping or nearby (within 1 million base pairs) the NDD CNV regions (Supplementary Table 8), finding that no gene-set remained significant after this adjustment (all FDR *q* > 0.05).

### Comparisons with common variant genetics

We generated PTSD polygenic risk scores for our data based on the recent independent MVP PTSD GWAS. We included PRS and cumulative NDD CNV carrier burden in a regression model of PTSD symptoms. PTSD PRS was significantly associated with increased PTSD symptoms (beta = 0.011, SE = 0.0004, *p* = 9.8 × 10^−158^; OR = 1.16, 95%CI = [1.15,1.17]) and explained 0.5% of the total variation in PTSD symptoms (SE = 0.04%, *p* = 2.6 × 10^−33^). NDD CNV burden was also significantly associated with PTSD symptoms (beta = 0.0287, SE = 0.0053, *p* = 7.7 × 10^−8^; OR = 1.57, 95%CI = 1.31,1.89), and explained an additional 0.034% (SE = 0.0001, *p* = 0.0017) of the variation in PTSD symptoms.

## Discussion

We identified an association between the cumulative burden of CNVs and PTSD, which was largely driven by CNVs overlapping previously implicated NDD CNV regions. Two recent studies of CNVs in major depression [9, 10] also reported associations with NDD CNV burden, with effect sizes comparable to ours. The modest to moderate effect sizes observed are consistent with PTSD and MDD being disorders with less severity of cognitive impairment, comparatively moderate heritability and a larger environmental component. In terms of how CNV burden modifies depression risk, Kendall et al. [9] suggested that the majority of the total effect came from the direct effects of CNVs, with some evidence of additional mediated effects stemming from sociodemographic risk factors including physical health, smoking, alcohol consumption, educational attainment, and social deprivation. As PTSD has similar risk factors [31], NDD CNVs may influence PTSD risk via the same mediated mechanisms. We propose that some of the psychiatric and neurodevelopmental consequences of CNVs may also increase PTSD risk, as they represent PTSD risk factors [32] [33].

In examining the individual NDD CNVs, we observed a significant association of PTSD with the 15q11.2 BP1-BP2 microdeletion, one of the most frequently occurring pathogenic CNVs identified in humans [34]. This CNV is associated with alterations in brain morphology and cognition [35]. There is a wide variety of possible clinical manifestations, including developmental delays, intellectual disability, as well as behavioral and psychiatric problems, including ADHD, ASD and schizophrenia [36]. Under a less strict definition of NDD carrier status (>0% overlap with NDD CNV region), the 22q11.2 duplication region and 8p23.1 deletion regions were significant. The 22q11.2 duplication has a variety of deleterious impacts [37], but generally they are less severe than those observed in the 22q11.2 deletion [38]. The 8p23.1 deletion is associated with developmental delays, hyperactivity, and impulsivity [39]. Rather than any specific functional aspects of these CNVs having led to the significant associations that we observed, we suspect that their relatively high frequencies in the data made them among the most statistically powered to identify.

Pathway specific enrichment of brain regions and neurodevelopmental gene-sets has consistently been observed in genetic studies of psychiatric disorders [3, 40–42]. We have identified significant associations with several biological pathways related to the development of the brain and nervous system. Our pathway analysis was not significant once we removed the CNV overlapping NDD regions, possibly suggesting an outsized or central role of genes in NDD regions relative to other genes within the pathways. Genes in NDD regions are known affect the development of the brain and nervous system, likely through the disruption of core molecular pathways [43] [44].

The regions we have identified as significant in CNV analyses have not been implicated in GWAS of PTSD. These regions may represent a distinct element of the genetic contribution to PTSD risk that is not readily identified by common variant analyses, suggesting that rare variation analysis complements common variant analysis, as has been hypothesized for psychiatric phenotypes [4]. The effects of implicated CNVs were modest in magnitude, albeit higher than reported common variant effects, consistent with the hypothesis that rare variants have stronger effects than common ones [45].

In terms of population risk prediction, due to the limited number of CNV carriers, CNV burden predicted substantially less total variation in PTSD than PRS. The utility of determining carrier status, rather than population level prediction, is that CNV carriers may be a subset of individuals for whom a tailored health management strategy [46] applies. Indeed, CNV carrier status has been proposed as a tool in clinical decision making for psychiatric disorders, albeit one that will first require expansion of the clinical knowledge base of CNVs [47]. But it is unclear how much this will apply directly to PTSD, as we did not identify any highly penetrant CNVs.

### Limitations

We focused only on rare (<1% frequency) CNVs larger than 10 kilobases in length due to the detection limits of array based CNV calling. However, small CNVs may have clinical importance [48, 49]. Future investigation of the relationship between small CNVs and PTSD will likely require sequencing data, as the dense genotyping allows for the determination of CNV at a higher resolution than SNP genotyping arrays [50]. Thus we expect that CNV investigations will emerge as sequencing data becomes available from biobank resources [51]. We were unable to assess the impact of de novo CNV specifically, which would require case-parent trio data to identify. Yet, de novo variation is an important form of risk to investigate, as it occurs more often in cases than controls for ADHD, ASD, and schizophrenia [52]. PTSD genetic studies usually do not gather parent genotype data, implying that new data would need to be gathered in order to study this. We note that several of the cohorts investigated were from specially selected populations. The UKBB is known to be healthier than the general population of the United Kingdom [53]. As well, we analyzed several military populations, where good physical and mental health are required for enlistment. Due to carriers not having been selected for health reasons consequential to their carrier status, our study may have incorrectly estimated (or outright not detected) some effects of CNV on PTSD. Indeed, this may be why we specifically identified the 15q11.2 BP1-BP2 deletion and 22q11.2 duplication: As these CNVs have relatively milder impacts compared to some CNVs [54] [38], more seemingly unaffected carriers would exist in the investigated cohorts. We did not identify any particular genes where the presence of CNVs had a significant association with PTSD. The limited statistical power of low frequency variation [55] perhaps inhibited our ability to detect these genes. Therefore, we hypothesize that specific gene associations will emerge given greater sample sizes or analytic techniques more suited for this form of data, especially as we had positively identified specific gene-sets. We only tested for enrichment of gene sets related to the brain and nervous system, however, CNV may act on other relevant pathways; CNV are thought to have widespread phenotypic effects, such as on the immune system [56], which is also deeply implicated in PTSD development [57]. We did not examine non-European ancestry populations owing to insufficient sample sizes, but there is a clear need to include them in genetic research studies [58]. Collection of such samples is an ongoing aim of the PGC-PTSD [2].

## Conclusions

We have performed, to our knowledge, the largest (*N* = 114,383 participants) investigation of the influence of CNV burden on PTSD risk, and furthermore, are the first to identify significant associations. Risk was enriched in regions that crossed over known NDD regions. In particular, we have implicated the 15q11.2 BP1-BP2 microdeletion. Larger sample size data, better detection methods, and annotated resources of CNV are necessary to explore these relationships further.

## Supplementary information

Supplementary figure legends

Supplementary Figure 1

Supplementary Table 1

Supplementary Table 2

Supplementary Table 3

Supplementary Table 4

Supplementary Table 5

Supplementary Table 6

Supplementary Table 7

Supplementary Table 8

## Data Availability

Code is available from https://github.com/nievergeltlab/cnv_freeze1 or by request to the corresponding author.
