# Supplementary material for: Rare copy number variation in posttraumatic stress disorder
Source: Mol Psychiatry. Author manuscript; Available in PMC 2022 Dec 22. (PMC9763110; doi:10.1038/s41380-022-01776-4)
Supplement: Supplementary Figure Legend [file NIHMS1853619-supplement-Supplementary_Figure_Legend.docx]

Supplementary Figure Legends

SFig1. Histogram of the distribution of CNV length across the data. The x-axis depicts log (base 10) transformed CNV length data (in megabases). The Y axis is the frequency observed in the data.
