# Supplementary figures and images for: Rare copy number variation in posttraumatic stress disorder
Source: Mol Psychiatry. Author manuscript; Available in PMC 2022 Dec 22. (PMC9763110; doi:10.1038/s41380-022-01776-4)

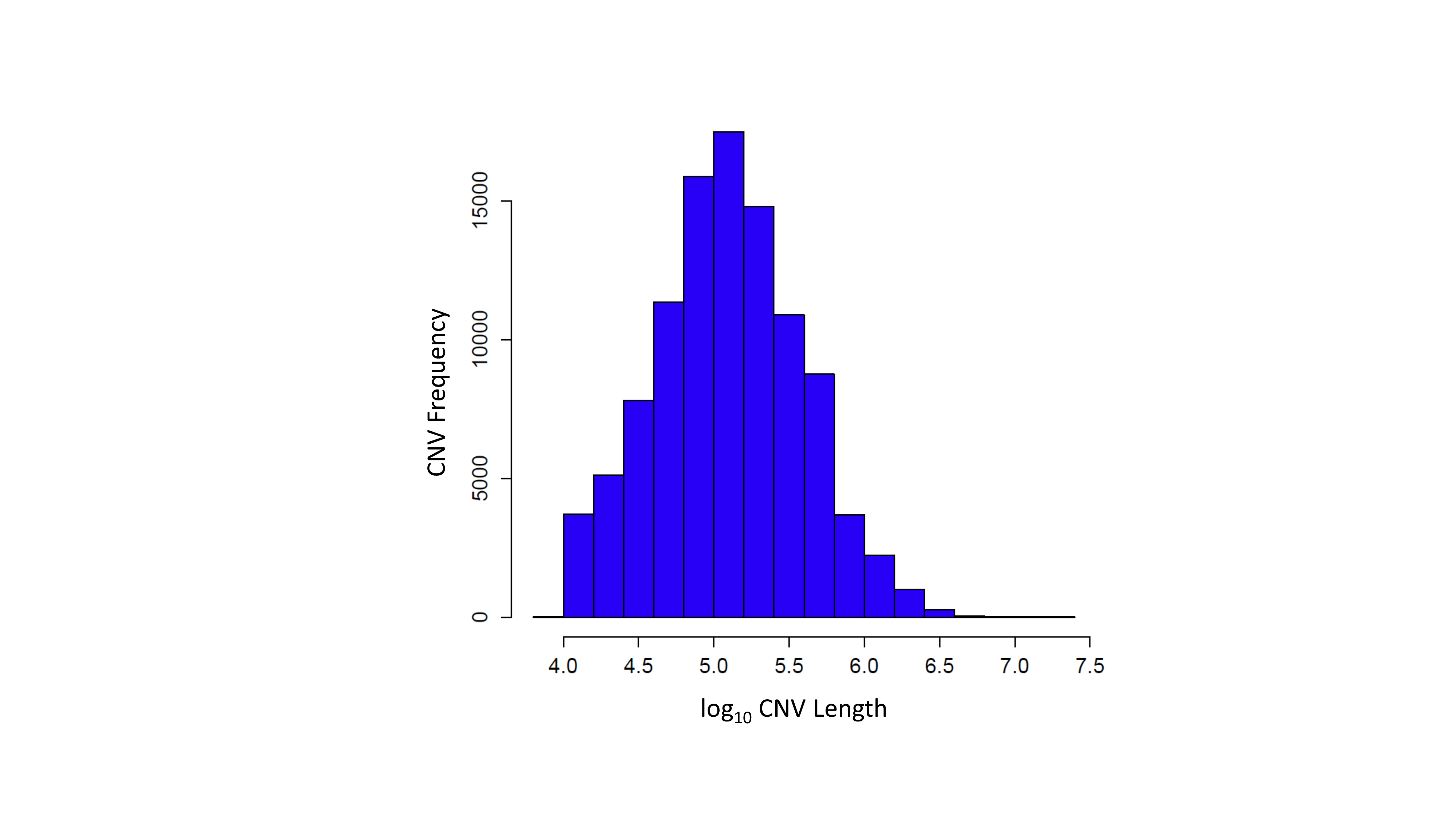

Supplement: Supplementary Figure 1 [file NIHMS1853619-supplement-Supplementary_Figure_1.png]
